# A ying and yang balance: transcription factors OsNAC2 and OsEREBP1 synergistically regulate plant immunity

**DOI:** 10.1093/plphys/kiae031

**Published:** 2024-01-19

**Authors:** Yuan Fang, Jian Chen

**Affiliations:** College of Chemistry and Life Sciences, Zhejiang Normal University, Jinhua 321004, China; Assistant Features Editor, Plant Physiology, American Society of Plant Biologists; International Genome Center, Jiangsu University, Zhenjiang 212013, China

Rice (*Oryza sativa*) is one of the most important staple foods that feeds more than half of the global population. Its yield is seriously threatened by diseases caused by bacteria, fungi, and viruses. One of the most devastating bacterial pathogens, *Xanthomonas oryzae* pv. *oryzae* (*Xoo*), causes bacteria blight, resulting in reduced rice yield and quality (Mansfield et al. 2012). Therefore, it is of great importance to understand the disease resistance mechanism and to identify resistance genes for global food security.

Transcription factors play important roles in plant growth, development, and disease resistance. Of these, the NAC (NAM-ATAF-CUC) family of transcription factors has been extensively studied in response to various stresses, including heat, cold, drought, flood, salt, and disease stresses (Han et al. 2023). Previous studies showed that OsNAC2 plays important roles in biological processes, including root growth and development, leaf senescence, and abiotic stress tolerance (Mao et al. 2017; Shen et al. 2017; Mao et al. 2020). What is the role of OsNAC2 in biotic stress?

In this issue of *Plant Physiology*, Zhong et al. characterized the function of OsNAC2 in plant disease response and found that it functions as a negative regulator in the resistance to bacterial blight disease in rice (Zhong et al. 2023). To determine the role of OsNAC2 in bacterial blight disease in rice, Zhong et al. initially examined the expression levels of *OsNAC2* in rice leaves inoculated with *Xanthomonas oryzae* pv. *oryzae* (*Xoo*) strain PXO71 and found that it was significantly induced. This finding suggests that OsNAC2 plays a role in disease resistance to *Xoo*. Indeed, *OsNAC2*-overexpressing lines were more susceptible to *Xoo* compared with wild-type plants, whereas the RNAi lines displayed increased disease resistance. Consistently, the accumulation of H_2_O_2_ and O_2_^−^ and the expression of disease marker genes *PR1a* (*Pathogenesis-related protein 1a*) and *PR10a* were suppressed by OsNAC2.

As salicylic acid plays an important role in plant disease resistance (Chen et al. 2021), the authors investigated whether OsNAC2 regulates SA production and perception (Zhong et al. 2023). Gene expression analysis showed that SA biosynthesis gene *OsICS1* (*isochorismate synthase 1*) and SA receptor gene *OsNPR1* (*nonexpressor of pathogenesis-related genes 1*) were sharply increased in the RNAi lines compared with WT. Furthermore, the authors found that OsNAC2 bound to the promoter of *OsICS1* and *OsNPR1* and suppressed their promoter activities. These findings suggest that OsNAC2 negatively regulates the expression of the SA biosynthesis and receptor genes (Fig).

**Figure 1.**
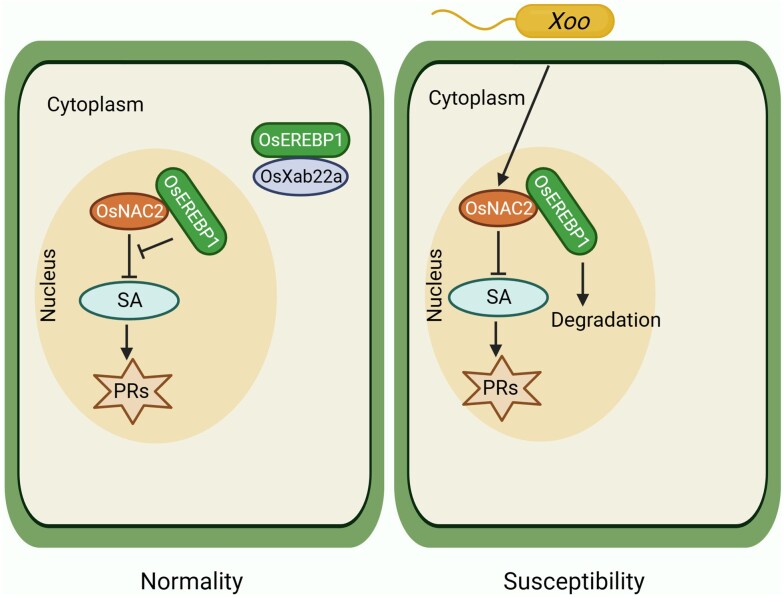
A schematic model shows the OsNAC2-OsEREBP1 regulation module. Under normal conditions, OsEREBP1 localizes both in the cytoplasm and nucleus to maintain a steady-state balance. Upon *Xoo* infection, the expression of *OsNAC2* was induced, resulting in stronger inhibition of the SA signaling pathway. OsEREBP1 was imprisoned in the nucleus for degradation, disrupting the homeostasis and causing plant susceptibility.

To further understand the function of OsNAC2, Zhong et al. conducted a screening of potential OsNAC2 interactors and identified one protein of particular interest called OsEREBP1 (ethylene response element binding protein 1), which belongs to the AP2/ERF (APETALA2/ethylene response factor) family. According to a previous report by Jisha et al. (2015), OsEREBP1 acts as a positive regulator of rice resistance bacterial blight (Jisha et al. 2015). Zhong et al. found that OsNAC2 interacted with OsEREBP1 in the nucleus, forming a complex that co-regulated the expression of target genes. OsNAC2 alone suppressed the transcription of *OsICS1*, but this reduction was partially restored when OsNAC2 and OsEREBP1 were co-expressed (Zhong et al. 2023). It is widely recognized that rice immune receptor Xa21 confers resistance to *Xoo* (Park and Ronald 2012). Zhong et al. found that OsEREBP1 interacted with OsXb22a in the cytoplasm. Furthermore, they found that OsXb22a was stabilized by OsEREBP1. However, in *OsNAC2*-overexpressing lines, the accumulation of OsEREBP1 is more confined in the nucleus. Upon *Xoo* infection, OsEREBP1 in the nucleus was degraded in *OsNAC2*-overexpressing lines.

In summary, Zhong et al. discovered that OsNAC2 functions as a negative regulator in the resistance to bacterial blight disease in rice. OsNAC2 negatively regulates the expression of SA biosynthesis and receptor genes by binding to their promoters and suppressing their activities. To counteract the negative regulation of plant immunity by OsNAC2, OsEREBP1 interacts with OsNAC2 in the nucleus and disrupts the negative effect of OsNAC2 on the expression of SA biosynthesis genes (Fig). OsEREBP1 also interacts with Xa21-binding protein OsXb22a in the cytoplasm and stabilizes it to confer disease resistance. Thus, maintaining the homeostasis of OsEREBP1 in the cytoplasm and nucleus is important for rice resistance to *Xoo*. However, in OsNAC2 overexpression lines, OsEREBP1 is largely confined in the nucleus, resulting in disruption of OsEREBP1 homeostasis. Under this scenario, *Xoo* infection causes the reduced stability of OsEREBP1, leading to the development of disease. Overall, Zhong et al. unveiled the homeostatic regulatory role of OsNAC2-OsEREBP1 in rice immune response. Their findings provide insight into the molecular breeding of rice resistance to bacterial blight disease.
